# PagSOD2a improves poplar salt tolerance by elevating superoxide dismutase activity and decreasing malondialdehyde contents

**DOI:** 10.3389/fpls.2024.1456249

**Published:** 2024-09-13

**Authors:** Lieding Zhou, Changhong Yu, Siyuan Nan, Yajing Li, Jia Hu, Kai Zhao, Jinping Guo, Shengji Wang

**Affiliations:** College of Forestry, Shanxi Agricultural University, Jinzhong, China

**Keywords:** poplar, SOD gene, salt stress, superoxide anion, gene network

## Abstract

Superoxide dismutase (SOD) is widely present in plants and plays a crucial role in defending against oxidative stress and preventing tissue damage. This study discovered that the *PagSOD2a* gene in 84K poplar (*Populus alba* × *P. glandulosa*) exhibits a distinct capacity to be induced in response to salt stress. To delve into the pivotal role of *PagSOD2a* in conferring salt tolerance, the entire *PagSOD2a* fragment was successfully cloned from 84K poplar and the potential function of *PagSOD2a* was explored using bioinformatics and subcellular localization. *PagSOD2a* was found to encode a CuZn-SOD protein localized in chloroplasts. Furthermore, six CuZn-SOD family members were identified in poplar, with closely related members displaying similar gene structures, indicating evolutionary conservation. Morphological and physiological indexes of transgenic 84K poplar overexpressing *PagSOD2a* (OE) were compared with non-transgenic wild-type (WT) plants under salt stress. The OE lines (OE1 and OE3) showed improved growth performance, characterized by increased plant height and fresh weight, along with reduced malondialdehyde (MDA) content and electrolyte leakage rate under salt stress. Meanwhile, overexpression of *PagSOD2a* significantly augmented CuZn-SOD and total SOD enzyme activities, leading to a reduction in superoxide anion accumulation and an enhancement of salt tolerance. Additionally, co-expression and multilayered hierarchical gene regulatory network (ML-hGRN) mediated by *PagSOD2a* constructed using transcriptome data revealed that *PagSOD2a* gene may be directly regulated by *SPL13*, *NGA1b* and *FRS5*, as well as indirectly regulated by *MYB102* and *WRKY6*, in response to salt stress. These findings provide a theoretical and material foundation for further elucidating the function of *PagSOD2a* under salt stress and for developing salt-tolerant poplar varieties.

## 1 Introduction

At present, global climate change, along with the intensifying severity of soil salinization, has made salt stress one of the major challenges impacting the growth and development of plants. Studies have indicated that the global area of salinized land is nearly 954 million hectares (Wang et al., 2017), which adversely affects plant growth and crop yields. The impact of salt stress on plants is mainly manifested by the hindrance of water absorption due to excessive salts, leading to cellular dehydration and ultimately affecting normal plant growth and development (Munns and Tester, 2008). Simultaneously, salt stress leads to an increase in the levels of reactive oxygen species (ROS) within plants, which can react with intracellular lipids, proteins, and nucleic acids, to trigger lipid peroxidation, protein denaturation, and nucleic acid damage. Without timely scavenging, these effects may damage cellular structure and function or even cause cell death, posing a serious threat to plant survival (Bose et al., 2013).

To mitigate these ROS, plants have evolved complex antioxidative defense systems comprising a series of enzymatic antioxidative mechanisms as well as non-enzymatic antioxidants. Enzymatic antioxidants such as superoxide dismutase (SOD), peroxidase (POD), glutathione peroxidase (GPX), and catalase (CAT) are important components of the plant’s antioxidative defense system (Alscher et al., 2002). Among them, SOD is a widely existing enzyme responsible for converting harmful superoxide radicals (O_2_^-^) into oxygen (O_2_) and hydrogen peroxide (H_2_O_2_), the latter of which can be further transformed and decomposed by antioxidative systems like CAT and GPX, hence maintaining the cell’s redox homeostasis (Mittova et al., 2000). Through this catalytic action, SOD enzymes are critical in resisting oxidative stress and preventing tissue damage (Zelko et al., 2002). SOD enzymes can be classified into several types based on their metal cofactors. The three main types are copper/zinc SOD (CuZn-SOD), manganese SOD (Mn-SOD), and iron SOD (Fe-SOD) (Wuerges et al., 2004). CuZn-SOD primarily resides within the cytoplasm and mitochondria-intermembrane spaces of eukaryotes, with a structure consisting of a *β*-barrel that holds a copper ion and a zinc ion. Mn-SOD and Fe-SOD are mainly located in the mitochondria and some prokaryotic organisms, with manganese and iron ions bound, respectively. Though they share similar tertiary structures, their catalytic mechanisms are different. Beyond these primary types, studies have also identified nickel-containing SODs (Ni-SOD) in a minority of bacteria (Alscher et al., 2002).

Research has shown that enhancing the expression of *SOD* in *Solanum lycopersicum* through genetic engineering under drought and salt stress conditions significantly strengthens the plant’s stress tolerance and optimizes growth performance (Shafi et al., 2017). Additionally, transgenic plants overexpressing *SOD* and *APX* enhanced lignification and starch biosynthesis, and improved salt tolerance (Sharma and Dietz, 2006; Shafi et al., 2015). Furthermore, the metabolic processing of heavy metals by SOD in plants is crucial that plant cells alleviate toxicity and damage from metals such as lead (Pb) or cadmium (Cd) through upregulating SOD activity (Suzuki and Mittler, 2006; Li et al., 2017). An increase in SOD activity in *Zea mays* under cold conditions demonstrates its key roles in resisting low-temperature stress (McKersie et al., 1993). In transgenic *Medicago sativa*, overexpression of *Nicotiana benthamiana Mn-SOD* enhanced tolerance to freezing stress (Che et al., 2020). Similarly, overexpressing potato *Cu/Zn-SOD* improved transgenic *S. tuberosum*’ tolerance in cold environments (Khanna-Chopra, 2012). In addition to play a protective role during environmental stress, SOD is also involved in the normal physiological process in plants via regulating ROS levels to help maintain cellular function (Zhou et al., 2023). However, researches on CuZn-SOD members in woody plants is scarce and existing studies suggest CuZn-SOD functions vary among different plants.

Poplars (*Populus* L.) are extensively used in biofuel production, road greening, and desertification prevention forests (Tuskan et al., 2006). With the establishment of genetic transformation systems as well as the availability of genomic database, poplars have become one of the most valuable model systems in woody plant biology research (Cseke et al., 2007; Wang et al., 2021a). Due to land reclamation and unsustainable irrigation practices, salt stress has emerged as a significant environmental stress factor limiting the growth and products of poplar trees (Tuskan et al., 2006). Therefore, exploring the regulatory mechanism of the *SOD* genes in poplars and breeding new poplar varieties with salt tolerance is of great significance. In this study, among the six CuZn-SOD family genes identified in poplar, *PagSOD2a* (homologous with *Potri.004G216700* of *P. trichocarpa*) was found to be significantly induced by salt stress, with its transcript levels being higher than those of the other poplar CuZn-SOD family members (**Supplementary Figure S1**). Subsequently, *PagSOD2a* was selected for further experiments, and a comparative analysis of morphological and physiological indices was conducted between *PagSOD2a* overexpressing transgenic lines (OE) and non-transgenic plants (WT) under salt stress conditions to elucidate the function of *PagSOD2a* under salt stress.

## 2 Materials and methods

### 2.1 Bioinformatic analysis

The protein sequences of poplar were downloaded from the Phytozome database (https://phytozome-next.jgi.doe.gov/, accessed on 5 July 2023) (Tuskan et al., 2006; Goodstein et al., 2012). Then, the Sod_Cu domain (PF00080) was obtained from the Pfam database (https://www.ebi.ac.uk/interpro/, accessed on 3 October 2023) and searched to identify the CuZn-SOD proteins using hmmsearch (http://www.hmmer.org/, accessed on 3 October 2023) with a threshold of E-value < 1×10^-5^ (El-Gebali et al., 2019). Members containing the conserved domain were selected and validated using Pfam and SMART databases (http://smart.embl-heidelberg.de/, accessed on 3 October 2023) (Letunic et al., 2015; Letunic and Bork, 2018; El-Gebali et al., 2019). After confirming the members of the CuZn-SOD gene family, the protein sequences of each member were retrieved from the Phytozome database (https://phytozome-next.jgi.doe.gov/, accessed on 7 October 2023) and were used to perform multiple sequence alignment by Bioedit 7.2.5 (Hall, 1999). A phylogenetic tree was constructed using MEGA 11.0.13 via the Neighbor-Joining method and 1,000 bootstrap replicates. Subsequently, the coding sequences and genomic sequences of the genes were downloaded from the Phytozome database (https://phytozome-next.jgi.doe.gov/, accessed on 7 October 2023) (Tuskan et al., 2006; Goodstein et al., 2012). The conserved motifs in the CuZn-SOD proteins were identified using the MEME Suite (https://meme-suite.org/meme/, accessed on 7 October 2023) (Bailey et al., 2009), and the annotated information file of poplar gene structure was downloaded from the Phytozome database (https://phytozome-next.jgi.doe.gov/, accessed on 5 July 2023). The identified motifs and intron/exon structures of CuZn-SOD members were visualized using the Gene Structure View (Advanced) in TBtools 2.086 (Chen et al., 2020).

### 2.2 Plant growth condition

The 84K poplar (*P. alba* × *P. glandulosa*) and *N. benthamiana* used in this study were grown on a 1/2 MS plant medium in a greenhouse (25 ± 2°C, 60–70% relative humidity, 16/8 h light/dark cycle, with supple-mental light of ~300 μEm^−2^s^−1^, three-band linear fluorescent lamp T5 28 W 6400 K). The roots, stems, and leaves used in this experiment were excised from the plants, immediately frozen in liquid nitrogen, and then stored at −80°C (Wang et al., 2021a).

### 2.3 Gene cloning and vector construction

Total RNA was extracted using RNAprep pure Plant Kit from plant leaves (TIANGEN, Beijing, China). cDNA was synthesized using FastKing gDNA Dispelling RT SuperMix (TIANGEN, Beijing, China). *PagSOD2a* was amplified using SOD2a-F/R primers (**Supplementary Table S1**) and ligated into the pMD19-T vector (Takara, Beijing, China) via TA cloning for sequencing. *PagSOD2a* without the stop codon was then amplified with the SOD2a-121-F/R primers (**Supplementary Table S1**) and inserted into the pBI121 vector via the *Sma I* and *Spe I* restriction sites to fuse GFP expression. Finally, the recombinant plasmid 35S:PagSOD2a-GFP was obtained.

### 2.4 Gene expression pattern analysis

To investigate the temporal expression pattern of the *PagSOD2a*, one-month-old tissue culture seedlings of 84K poplar with uniform statues were treated with 150 mM NaCl for 0 h, 3 h, 6 h, 12 h, 24 h and 36 h, respectively, with three replicates for each treatment. After the treatments, the roots, stems, leaves and buds of the plants were harvested and immediately frozen in liquid nitrogen. RNA was extracted from each sample for Real-time quantitative reverse transcription PCR (RT-qPCR) analysis with three technique repetition.

### 2.5 Subcellular localization

A 5 mL sterile needleless syringe was used to infiltrate tobacco leaves with *Agrobacterium tumefaciens* (GV3101) containing the GFP vector 35S:PagSOD2a-GFP or the empty vector 35S:GFP as control. After growing in the dark for 2-3 days, fluorescence signals were observed and photographed under a confocal laser scanning microscope (Olympus FV1000, Olympus, Tokyo, Japan) from different fields of view (Zhou et al., 2023).

### 2.6 Generation and identification of transgenic poplars

Transgenic poplars were obtained through *A. tumefaciens*-mediated transformation (He et al., 2018). In short, the 5^th^ and 6^th^ mature leaves of one-month old poplar seedlings were isolated and infected with the GV3101 solution at an OD600 of 0.6 for 10 mins, using the LB liquid medium without the addition of AS (acetosyringone). During this period, the leaves were shaken at 3-minute intervals to facilitate contact between the bacterial solution and the leaves. Then, the leaves were positioned on a differentiation medium containing 60mM kanamycin for differentiation culture. DNA and RNA were extracted from the leaves of the transgenic plants to detect the positive lines using PCR and RT-qPCR, respectively. Primers PagSOD2a-F/R (**Supplementary Table S1**) were employed to determine the relative expression of *PagSOD2a* in each line using SuperReal PreMix Plus (SYBR Green, TIANGEN, Beijing, China), with *Actin* as the internal reference gene. The fold change in gene expression was calculated using the 2^−ΔΔCT^ method (Livak and Schmittgen, 2001).

### 2.7 Salt tolerance assay

Salt tolerance assays were conducted using both transgenic poplars and WT plants. Three-month-old, uniformly grown, and robust plants from each line were selected for the experiments. The plants were subjected to either 0 mM or 150 mM NaCl solutions for a duration of 15 days. After the treatment period, plant height and fresh weight were measured and recorded. Various plant tissues were then harvested, and root systems were scanned and imaged using an EPSON EXPRESSION 10000 XL root scanning system (with a resolution set at 400 dpi) to measure primary root length, root diameter, root area, and root volume. The data were analyzed using WinRHIZO 2016p software (Regent Instruments Inc., Quebec, QC, Canada) (Wang et al., 2021b; Zhou et al., 2023).

### 2.8 Measurement of xylem fiber

Xylem fibers were isolated from the 10th internode of three-month-old poplar stems. Initially, the epidermis and phloem were carefully removed from the stem segments to obtain an adequate amount of xylem fiber material. The collected material was then subjected to a dissociation process using a xylem fiber dissociation solution (Lourenço et al., 2016). After thoroughly washing away the residual dissociation solution, the isolated xylem fibers were stained using a 1% safranin solution. The length of the stained xylem fibers was subsequently observed and measured under a microscope (Leica DM6B).

### 2.9 Histochemical staining and physiological index determination

Transgenic and WT lines were subjected to treatments with either 0 mM or 150 mM NaCl for 15 days, with the 0 mM NaCl treatment serving as the control. Then, 4^th^ leaves were excised and stained using nitroblue tetrazolium chloride (NBT) as described in previous studies (Kim et al., 2003; Zhao et al., 2019). The remaining leaves were utilized for the determination of various physiological indices. The total SOD activity, Copper-zinc superoxide dismutase (CuZn-SOD) activity, POD activity, proline content, and MDA content were measured using commercially available assay kits from Jiancheng Bioengineering Institute (Nanjing, China) following the instructions. Additionally, O_2_^-^ content was quantified using an O_2_^-^ content determination kit from Solarbio Science and Technology (Beijing, China). Electrolyte leakage rate was assessed according to the method described by Fan (Fan et al., 1997).

### 2.10 Multilayered hierarchical gene regulatory network

Transcriptome data of 84K poplar under salt stress were analyzed using the Majorbio Cloud Platform (https://cloud.majorbio.com). Leaves (second) from one-month-old WT plants were used for RNA-seq. WT plants were treated with 100 mM NaCl solution (WT_S) for 24 h. Sequencing was performed on 3 replicates of WT samples and 3 replicates of WT_S samples (each replicate consisting of material from 6 plants per treatment) using the Illumina NovaSeq 6,000 platform with 2 × 150 bp paired-end reads. The sequencing services were provided by Majorbio Biomedical Technology Co., Ltd. (Shanghai, China). The sequencing methods and differential expression gene screening followed the studies conducted by Wang and Huang (Huang et al., 2020; Wang et al., 2021a). A gene set comprising differentially expressed genes and *PagSOD2a* was created, and expression correlation analysis (coefficient = 0.5 and adjusted *P*-value < 0.05) was conducted using the Spearman method. KEGG pathway enrichment analysis of the co-expressed genes of *PagSOD2a* was performed using the online tools provided by the Majorbio Cloud Platform, employing Fisher’s exact test, with significant enrichment determined at an adjusted *P*-value < 0.05. The multilayered hierarchical gene regulatory network (ML-hGRN) was constructed using the BWERF method as described by Sisi Chen et al. and visualized using Cytoscape 3.10.2 (Shannon et al., 2003; Chen et al., 2024).

### 2.11 Statistical analysis

The data were processed using SPSS 22.0. Statistical significance between experimental and control groups was determined using a *p*-value threshold of < 0.05. Plots were generated using OriginPro 2021 and Adobe Photoshop 2022. Each experiment included three technical replicates.

## 3 Results

### 3.1 Phylogenetic tree and sequence structure analysis

**Figure 1:**
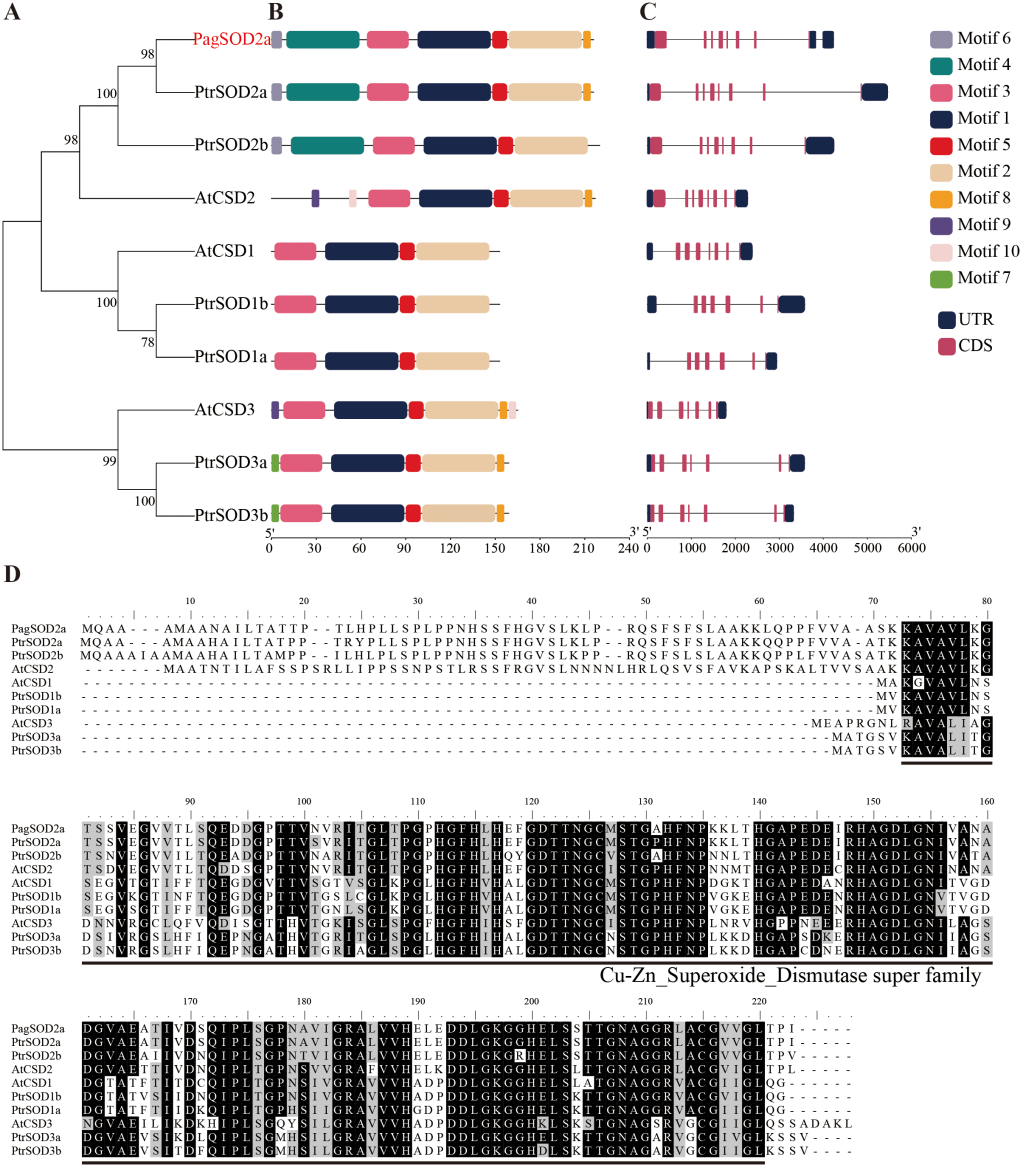
Comparative analyses of the phylogenetic relationships, protein conserved motifs, and gene structures of CuZn-SOD family in *Arabidopsis* and poplar. **(A)** Phylogenetic tree of 10 CuZn-SODs. **(B)** Motif composition of CuZn-SODs. The motifs are displayed by different-colored boxes. **(C)** Gene structures of *CuZn-SOD* genes. The black lines represent the introns, while the black and red boxes represent the untranslated regions (UTRs) and exons, respectively. **(D)** Multiple alignment of CuZn-SODs amino acid sequences. Black indicates identical amino acids and grey indicates similar amino acids. *PtrSOD2a*: *Potri.004G216700.4*; *PtrSOD2b*: *Potri.009G005100.3*; *PtrSOD1b*: *Potri.013G031100.9*; *PtrSOD1a*: *Potri.005G044400.5*; *PtrSOD3a*: *Potri.013G056900.2*; *PtrSOD3b*: *Potri.019G035800.2*; *AtCSD2*: *AT2G28190*; *AtCSD1*: *AT1G08830*; *AtCSD3*: *AT5G18100.1*.

The hmmsearch alignment results were validated using the Pfam and SMART databases, resulting in the identification of six members of the poplar CuZn-SOD family (**Figures 1A, D**). *PagSOD2a*, a homologous gene of *PtrSOD2a* (*Potri.004G216700*) in *P. trichocarpa* cloned from 84K poplar, was found to be significantly induced by salt stress compared to the other members of the poplar CuZn-SOD family (**Supplementary Figure S1**). Given the results obtained, *PagSOD2a* was chosen for further investigation due to its noteworthy response to salt stress, suggesting its potential significance in this particular context. MEME analysis illuminated that the CuZn-SOD family members possess 10 conserved motifs, with motifs 1, 2, 3, and 5 constituting fragments that are belong to the CuZn-SOD domain (**Figure 1B**). However, Motifs 4 and 6 were found only in PagSOD2a and its closest homologs, PtrSOD2a and PtrSOD2b, potentially indicating a specific biological function. Additionally, the intron-exon structure of the poplar CuZn-SOD members was examined (**Figure 1C**). These members possess 7 to 8 exons, and closely related members share similar gene structures, suggesting evolutionary conservation. Multiple sequence comparisons revealed that these proteins all possess a conserved CuZn-SOD_Dismutase super family structural domain, indicating their membership in the CuZn-SOD family (**Figure 1D**).

### 3.2 Analysis of gene expression patterns

**Figure 2:**
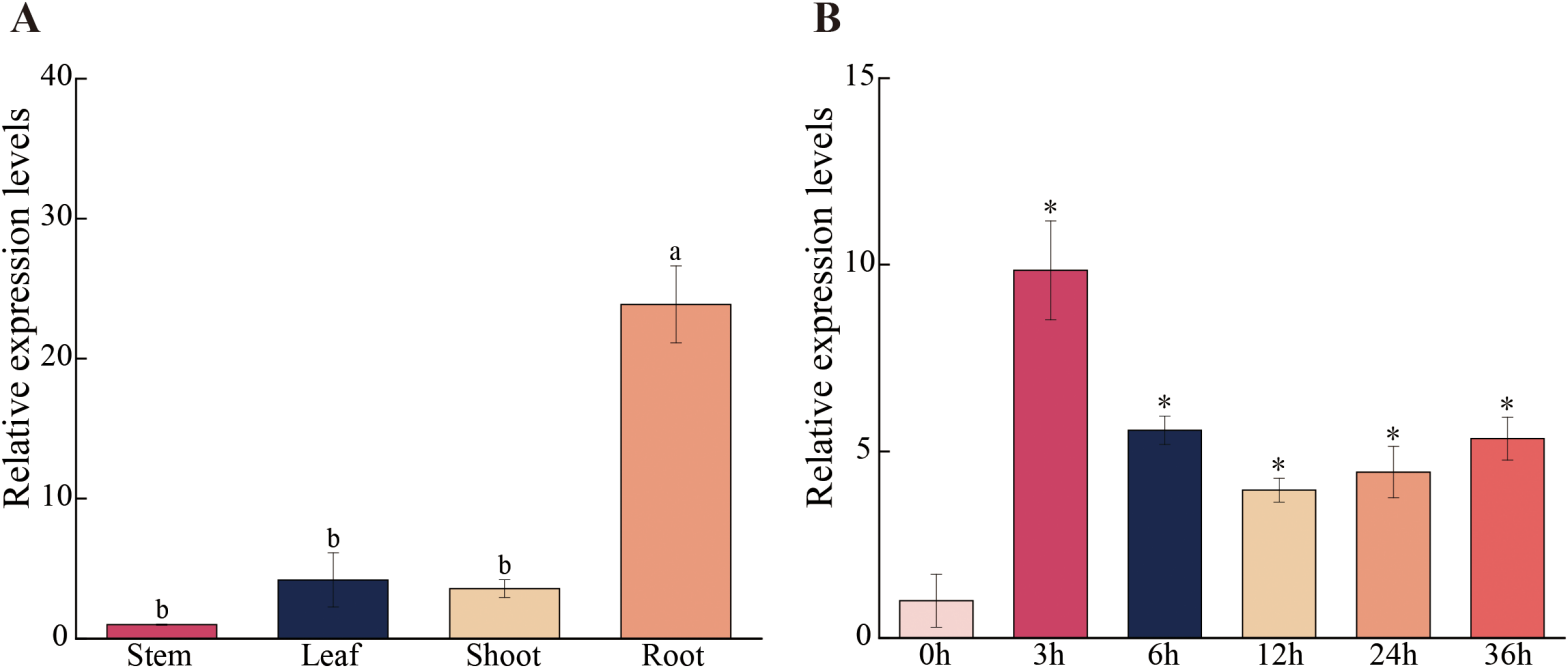
*PagSOD2a* gene expression pattern. **(A)** Expression levels of *PagSOD2a* gene in various tissues. The expression level of *PagSOD2a* in the stem is used as a reference for calculating the expression levels in other tissues; the error bar represents the standard deviation; different letters represent significant differences (P<0.05). **(B)** Temporal expression pattern analysis of *PagSOD2a* gene in the leaves of poplar under salt stress. Expression levels at other time points are calculated using the gene’s expression level at 0 h as the baseline; error bars represent standard deviation; * indicates significant differences compared to the expression level of the gene at 0 h (P < 0.05).

To investigate the differential expression patterns of the *PagSOD2a* gene in various tissues of 84K poplar, RT-qPCR was used to measure its expression levels in roots, stems, leaves and buds. The results, as shown in **Figure 2A**, indicated that the *PagSOD2a* gene has the highest expression levels in roots, followed by leaves and buds. The lowest expression level was observed in the stems.

Additionally, under salt stress conditions, *PagSOD2a* expression peaked at 3 hours after NaCl treatment and then decreased, but remained relatively high until the end of the treatment period (**Figure 2B**). This suggests that the *PagSOD2a* gene can rapidly respond to salt stress and actively participate in the poplar’s stress response throughout the duration of the salt treatment.

### 3.3 Localization analysis of PagSOD2a

**Figure 3:**
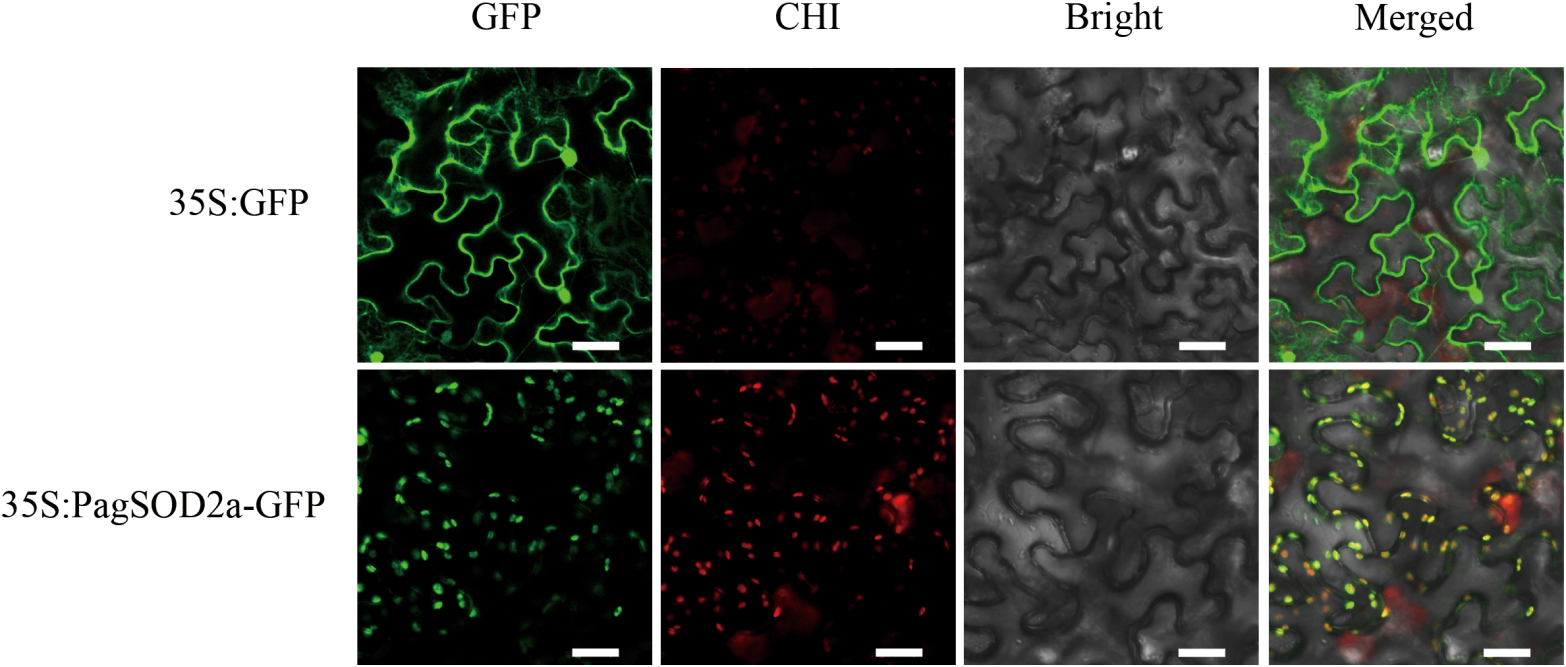
Subcellular localization of the PagSOD2a protein. The recombinant vector (35S:PagSOD2a-GFP) and the control vector (35S: GFP) were transiently expressed in tobacco leaves; GFP stands for green fluorescent protein; CHI represents chlorophyll fluorescence; Bright is the bright field; Merged is the overlay field; scale bar = 40µm.

The green fluorescent signal of 35S:PagSOD2a-GFP was predominantly localized in the chloroplasts of tobacco leaf cells, whereas the 35S:GFP signal was observed in the nucleus, cytoplasm, and cell membrane (**Figure 3**). These results indicate that PagSOD2a is a chloroplast-localized protein.

### 3.4 Generation and identification of transgenic plants

The 35S:PagSOD2a-GFP recombinant plasmid was transformed into 84K poplar using the *A. tumefaciens*-mediated leaf disk method, resulting in the cultivation of transgenic lines overexpressing *PagSOD2a*. Transgenic saplings that were able to root normally on 1/2 Murashige and Skoog (MS) medium supplemented with 50 mg/L Kanamycin and 200 mg/L Timentin were selected as positive clones (**Supplementary Figure S2**). The expression levels of *PagSOD2a* in four transgenic lines were measured using RT-qPCR. As shown in **Supplementary Figure S3**, compared with the WT line, *PagSOD2a* was significantly upregulated in two of the OE lines. The two lines with higher expression levels, OE1 and OE3, were selected for subsequent physiological experiments.

### 3.5 *PagSOD2a* enhances salt tolerance of poplar

**Figure 4:**
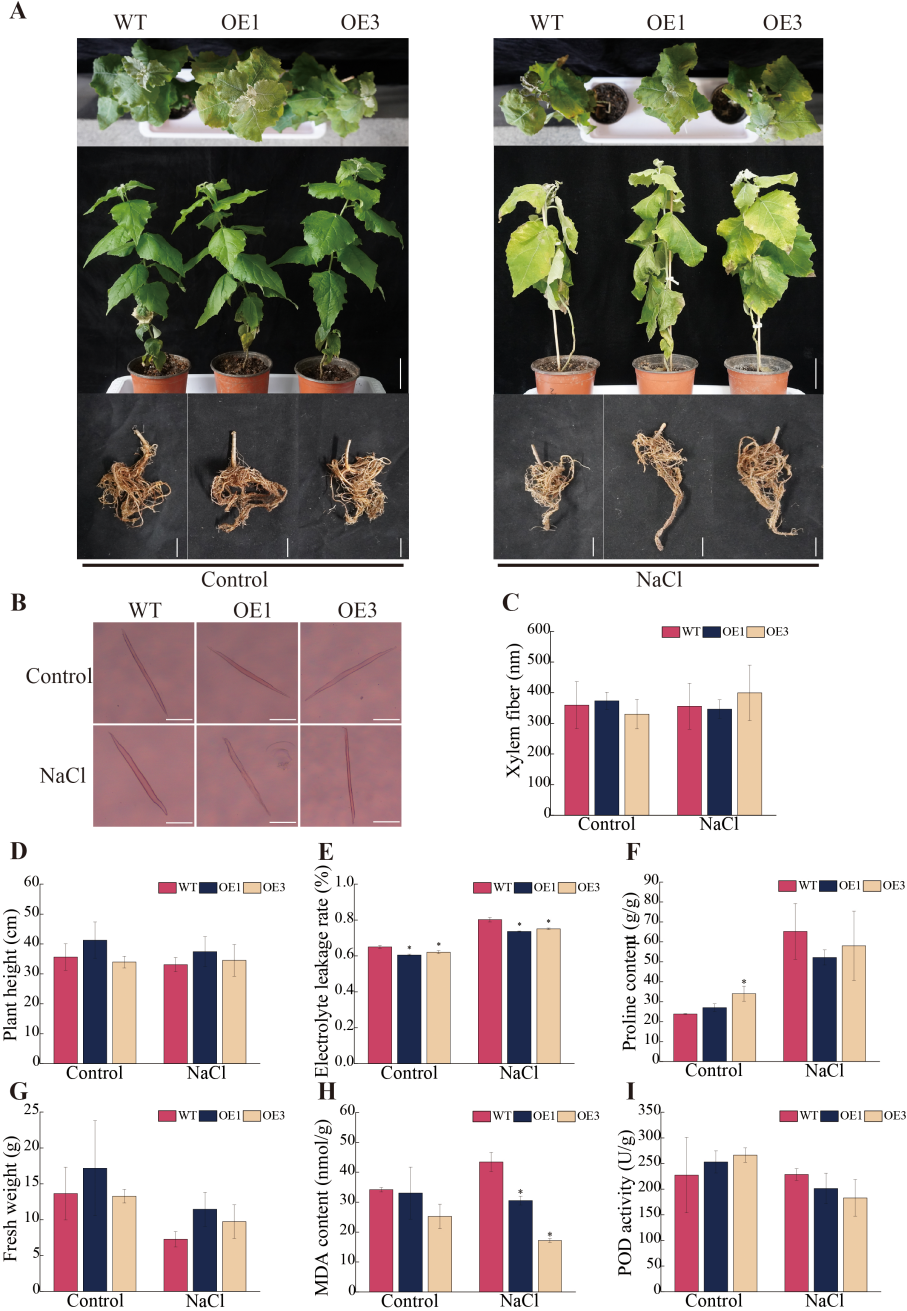
Stress tolerance analysis of *PagSOD2a* overexpression transgenic poplar. **(A)** Growth phenotype. **(B)** Morphology of xylem fibers. Scale bar: 100µm. **(C)** Statistical analysis of xylem fiber length; **(D)** Plant height. **(E)** Electrolyte leakage rate of each line under different treatments. **(F)** Proline content of each line under different treatments. **(G)** Fresh weight of above ground parts. **(H)** MDA content in each line under different treatments. **(I)** POD enzyme activity of each line under different treatments. Control is the water treatment control group, NaCl is the 150mM NaCl treatment group; WT is a non-transgenic line, OE1 and OE3 are lines overexpressing the *PagSOD2a* gene. The error bar represents the standard deviation. * indicates a significant difference between two samples (P < 0.05).

After 15 days of treatment with 150 mM NaCl solution, all lines experienced stress, with leaves turning yellow and wilting (**Figure 4A**). The shoot apices of WT line was the first to wilt, while the shoot apices of OE1 and OE3 lines remained viable. In the observation experiment on xylem fiber (**Figures 4B, C**), under control conditions, the xylem fiber length of the OE1 line was approximately 3.85% longer than that of the WT line, while the OE3 line had a xylem fiber length that was approximately 8.20% shorter than that of the WT line. Under salt stress conditions, the xylem fiber length of the OE1 line was approximately 2.63% shorter than that of the WT line, whereas the OE3 line exhibited a xylem fiber length approximately 12.28% longer than that of the WT line. All in all, *PagSOD2a* didn’t have much influence on the form of xylem fiber of poplar no matter under salt stress or not.

To investigate the specific effects of salt stress on the growth and development of each line, plant height and shoot fresh weight were first measured across different lines (**Figures 4D, G**). Under control conditions, there were no differences in plant height or shoot fresh weight among the different lines. After salt stress treatment, the plant height of OE1 and OE3 lines was approximately 13.09% and 4.33% higher than that of the WT line, respectively. The shoot fresh weight of OE1 and OE3 lines was approximately 57.43% and 33.68% higher than that of the WT line, respectively. However, there was no significant differences in root length, average root diameter, root area, or root volume (**Supplementary Figure S4**).

Subsequently, physiological indicators such as electrolyte leakage rate, MDA content, proline content, and POD enzyme activity were analyzed. The results showed that overexpression of the *PagSOD2a* gene significantly reduced electrolyte leakage and MDA content in OE leaves under salt stress (**Figures 4E, H**), indicating that the degree of cell membrane damage of OE was lower compared to WT line. Although proline content showed a significant increase in the OE3 line under control conditions, there was no significant difference under salt stress (**Figure 4F**). Additionally, the difference in POD enzyme activity was not significant (**Figure 4I**).

### 3.6 *PagSOD2a* enhances the O_2_^-^ scavenging capacity of poplar

**Figure 5:**
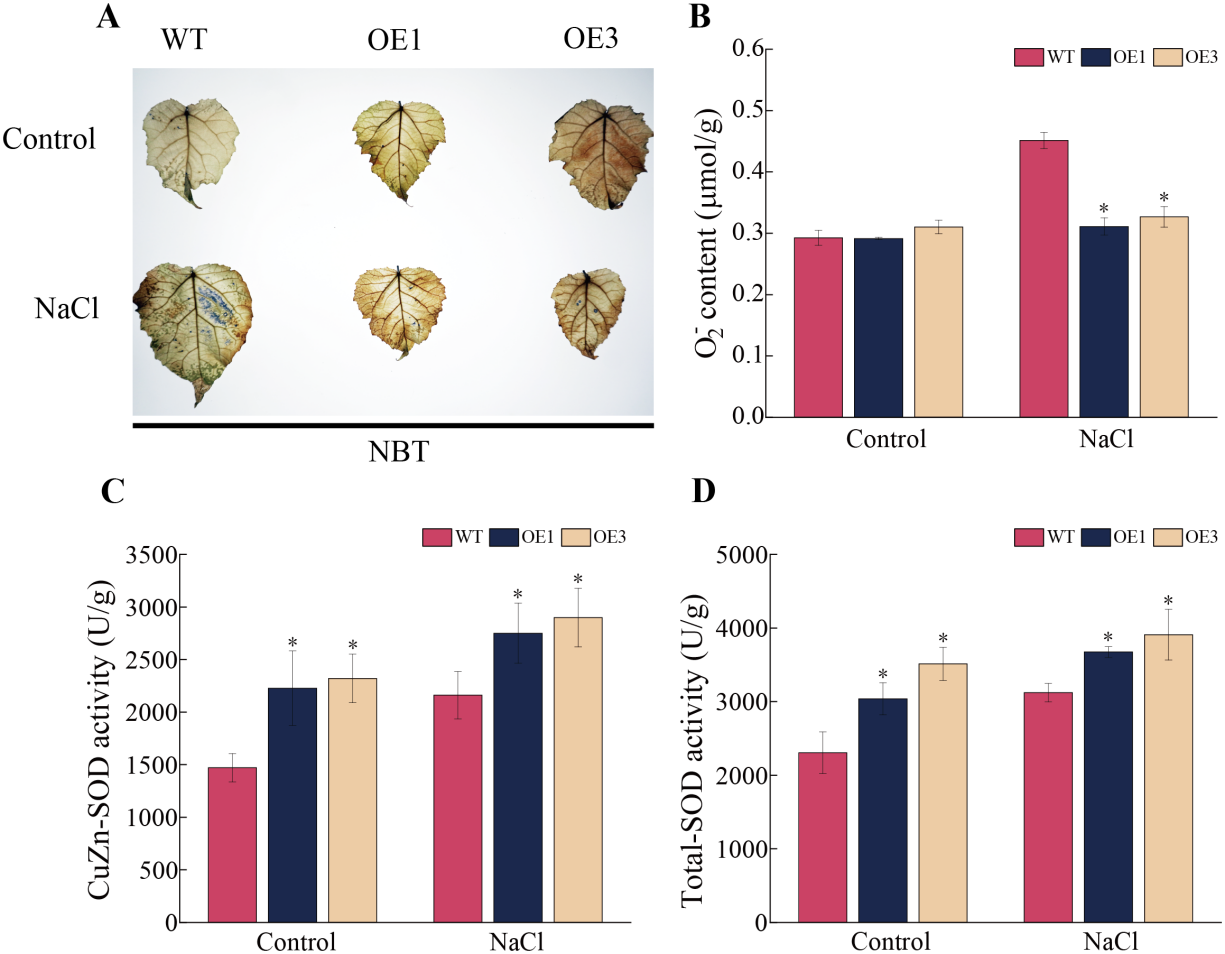
O_2_^-^ content and SOD enzyme activity of *PagSOD2a* overexpression transgenic poplar under different treatment. **(A)** NBT staining situation. **(B)** O_2_^-^ content. **(C)** CuZn-SOD enzyme activity. **(D)** Total SOD enzyme activity. Control is the water treatment control group, NaCl is the 150mM NaCl treatment group; WT is a non-transgenic line, OE1 and OE3 are lines overexpressing the *PagSOD2a* gene. The error bar represents the standard deviation; * indicates a significant difference between two samples (P < 0.05).

In plants, SOD enzymes can convert O_2_^-^ to O_2_ and H_2_O_2_, thereby reducing the risk of lipid peroxidation and cellular damage in the plant membrane, and enhancing the plant’s stress resistance. In this study, NBT staining and O_2_^-^ measurement indicated that, under control conditions, there were no significant differences in staining and O_2_^-^ content between the OE1 and OE3 lines and the WT line (**Figure 5A**). However, under salt treatment, the staining was lighter and had a smaller area in the OE1 and OE3 lines compared to the WT line. The average O_2_^-^ content in the OE1 and OE3 lines showed significant differences with approximately 31.10% and 27.64% lower than that in the WT line, respectively, (**Figure 5B**). The results of CuZn-SOD enzyme activity measurement showed that the CuZn-SOD and the total SOD enzyme activities in the OE1 and OE3 lines were significantly higher than that in the WT line under both control and salt stress conditions (**Figures 5C, D**). These results demonstrate that overexpression of the *PagSOD2a* gene can significantly increase the CuZn-SOD enzyme activity and total SOD enzyme activity in poplar, thereby reducing the accumulation of O_2_^-^ and enhancing salt tolerance of poplar.

### 3.7 *PagSOD2a* participates stress related pathways

**Figure 6:**
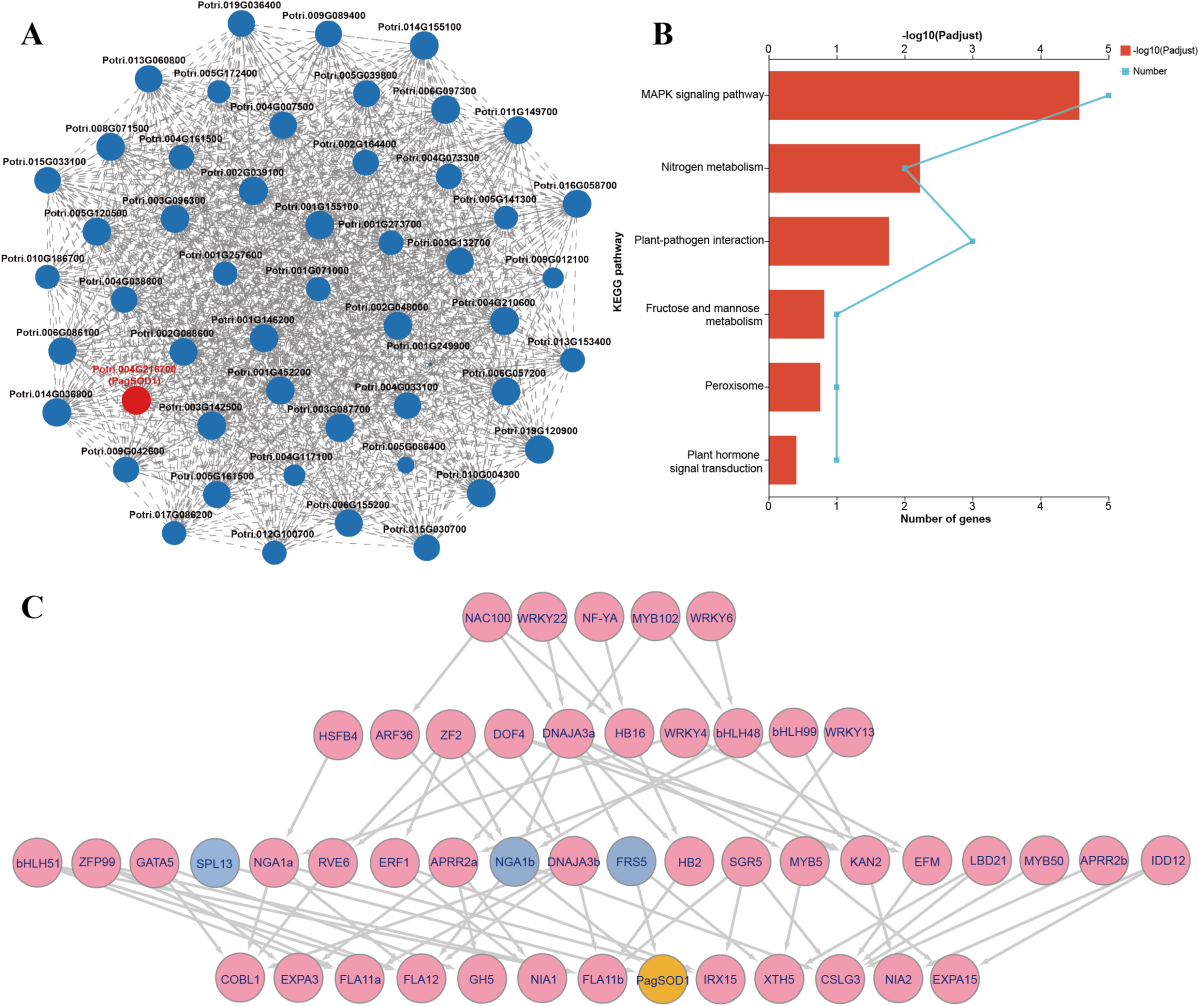
PagSOD2a participates stress related pathways via being regulated by transcription factors in different levels. **(A)** Genes co-expressed with *PagSOD2a* based on the RNA-seq data. Each node represents a gene, and the connections between nodes represent the correlation in expression between genes. The larger the node, the more genes it represents that have expression correlations with it. **(B)** KEGG enrichment analysis. **(C)** Multilayer hierarchical gene regulatory network. Yellow is *PagSOD2a*; Blue is the gene that directly regulates PagSOD2a; Pink is other co-expressed genes; The arrows represent the moderating relationships calculated by BWERF; The line width represents the im value.

Based on the RNA-seq data, a total of 50 genes showed expression correlations with *PagSOD2a*, among which 43 genes are directly related to *PagSOD2a* with a strong correlation, while 7 genes are indirectly co-expressed with *PagSOD2a* (**Figure 6A**). KEGG enrichment analysis revealed that the co-expressed genes of *PagSOD2a* were primarily enriched in the plant MAPK signaling pathway, nitrogen metabolism, and plant-pathogen interaction pathways, with additional enrichment in fructose and mannose metabolism, peroxisome, and plant hormone signal transduction pathways (**Figure 6B**).

Furthermore, a multi-layer hierarchical gene regulatory network was successfully constructed using the BWERF method. *PagSOD2a*, as a functional gene, was located at the bottom of the regulatory network and may be directly regulated by the third-tier transcription factors *SPL13* (*Potri.012G100700*), *NGA1b* (*Potri.001G452200*) and *FRS5* (*Potri.016G058700*) (**Figure 6C**). The second-tier transcription factors *ARF36* (*Potri.014G036800*), *ZF2* (*Potri.009G089400*), *DNAJA3a* (*Potri.006G097300*) and *bHLH48* (*Potri.006G057200*), and the first-tier factors *NAC100* (*Potri.017G086200*), *WRKY22* (*Potri.003G132700*), *MYB102* (*Potri.004G033100*) and *WRKY6* (*Potri.004G007500*) may indirectly participate in the regulation of *PagSOD2a*.

## 4 Discussion

Plants often produce excessive ROS when subjected to abiotic stresses such as high salinity, drought, cold, and heavy metal pollution. If these ROS are not promptly cleared, they can cause severe oxidative damage to cells, affecting the stability of biological macromolecules and the permeability of cell membranes (Zhang et al., 2016; Juan et al., 2021). ROS are usually normal byproducts of plant cell metabolism, but their production increases sharply under stress conditions (Apel and Hirt, 2004). Plants eliminate these ROS and maintain a balance between oxidation and reduction through a series of antioxidant enzymes. Among these, SOD intervenes early in the ROS scavenging mechanism, converting superoxide anions into O_2_ and H_2_O_2_, which are then converted into H_2_O by CAT or POD, thus reducing the toxic effects of ROS (McCord and Fridovich, 1969; Alscher et al., 2002). Numerous studies have shown that *SOD* genes can respond to various abiotic stresses. For example, in wheat and *Arabidopsis*, *TaSOD2* enhances salinity tolerance by regulating redox homeostasis through promoting NADPH oxidase activity (Wang et al., 2016). In rapeseed, overexpression of the *MnSOD* gene enhances tolerance to aluminum stress by increasing SOD enzyme activity (Basu et al., 2001). Similarly, overexpression of tamarisk *TaMnSOD* under salt stress reduces MDA content in poplar and increases SOD enzyme activity in transgenic poplar (Wang et al., 2010).

CuZn-SOD is one of the main members of the SOD family, widely present in higher plants, and actively participates in the plant response to abiotic stress. For instance, overexpression of the *Saussurea involucrata SikCSD* gene enhances tolerance to drought, low-temperature, oxidative, and salt stress in tobacco and cotton (Zhang et al., 2017, 2021). In rice, overexpression of the *OsCuZnSOD* gene similarly enhances salt tolerance (Guan et al., 2017). *CuZn-SOD* genes also play an active role in plant growth regulation and biomass synthesis. For example, under salt stress, overexpression of the *CuZn-SOD* and *APX* genes positively regulates secondary cell wall biosynthesis in *Arabidopsis*, promoting plant growth and yield (Shafi et al., 2015). Similarly, overexpression of the *CuZn-SOD* and *APX* genes not only enhances salt tolerance in transgenic potatoes but also increases lignin and starch biosynthesis (Shafi et al., 2017). These studies confirm the important role of *CuZn-SOD* genes in improving plant tolerance to salt stress.

In this study, we successfully cloned the *PagSOD2a* gene from 84K poplar, and our analysis revealed that the encoded protein is a member of the CuZn-SOD family. Previous studies have indicated that the CuZn-SOD family plays crucial roles in mitigating oxidative stress by scavenging superoxide radicals (Mishra and Sharma, 2008). Consistent with these findings, our results showed that the *PagSOD2a* gene shares evolutionary conservation with other CuZn-SOD family members, exhibiting similar gene structures among closely related members (**Figures 1A, D**). Our expression analysis revealed that *PagSOD2a* exhibits tissue-specific expression patterns, with the highest expression in roots, followed by leaves and buds, and the lowest in stems (**Figure 2A**). This finding aligns with previous studies suggesting that *SOD* genes often have tissue-specific expression patterns, contributing to their specialized functions in different plant parts (Feng et al., 2016). Under salt stress conditions, *PagSOD2a* expression peaked at 3 hours post-treatment but remained elevated thereafter, suggesting a potential role in early stress response mechanisms (Mittler et al., 2022). Such temporal expression patterns have also been observed in other *SOD* genes, indicating a complex regulatory network that modulates their expression under stress conditions (Feng et al., 2016; Yadav et al., 2019). Our subcellular localization experiments demonstrated that PagSOD2a is localized in chloroplasts (**Figure 3**), which is consistent with the essential role of chloroplasts in ROS metabolism and photooxidative stress management (Asada, 2006). This localization underscores the potential involvement of *PagSOD2a* in protecting chloroplasts from oxidative damage during salt stress. *PagSOD2a* expression was higher in roots, probably due to the presence of various non-chloroplast plastids in plant roots, such as amyloplasts, which are present in roots and can perform various essential functions, including starch storage, hormone synthesis, and stress responses (Köhler and Hanson, 2000; Natesan et al., 2005; Jarvis and Lopez-Juez, 2013; Quian-Ulloa and Stange, 2021). These non-chloroplast plastids may play a role in regulating the expression of *PagSOD2a* in roots. Physiological indicator analysis showed that *PagSOD2a* overexpressing lines had lower levels of MDA and electrolyte leakage rate under salt stress compared to WT line. Overexpression of the *PagSOD2a* gene significantly reduced the accumulation of O_2_^-^ by increasing CuZn-SOD and total SOD enzyme activities, thereby enhancing salt tolerance in poplar, though there were no significant differences in proline content and POD activity under salt stress, which might be due to their relatively independent roles in the salt stress response (Sharma et al., 2012).

Transcriptome data analysis of 84K poplar under salt stress revealed that 50 genes showed expression correlation with *PagSOD2a*, primarily enriched in plant MAPK signaling pathways, nitrogen metabolism, and plant-pathogen interaction pathways, suggesting that the *PagSOD2a* gene might be involved in plant growth and development processes, as well as responses to biotic and abiotic stresses. The multi-layer hierarchical gene regulatory network showed that *PagSOD2a* might be directly regulated by *SPL13*, *NGA1b*, and *FRS5*. Additionally, *MYB102* and *WRKY6* in the first tier might indirectly regulate *PagSOD2a* in responding to salt stress. *MYB102* enhances biotic stress sensitivity in *Arabidopsis* (Zhu et al., 2018) and may act in osmotic stress and wounding signal pathways (Denekamp and Smeekens, 2003), whereas *WRKY6* positively regulates ABA signaling in seed germination and early seedling development in *Arabidopsis* (Huang et al., 2016) and plays essential roles in senescence, pathogen defense (Robatzek and Somssich, 2002) and phosphate homeostasis (Su et al., 2015). This indicates that *PagSOD2a*, as a downstream gene of *MYB102* and *WRKY6*, may also participates in biotic and abiotic stress responses. All of the above results suggest the important roles of the *PagSOD2a* gene in plant response to abiotic stress and provide a theoretical basis for further elucidating the regulatory relationships in plant abiotic stress pathways, though specific regulatory mechanisms require further validation.

## 5 Conclusion

The results of this study highlight the significant role of the *PagSOD2a* gene in enhancing poplar’s salt tolerance. By elevating SOD activity and decreasing MDA contents, *PagSOD2a* contributes to reducing oxidative stress damage and maintaining better developmental states in poplar under salt stress. This provides a valuable theoretical and material basis for the cultivation of salt-tolerant poplar varieties, which is of great importance for sustainable commercial production of poplar. With the increasing challenges posed by soil salinization, developing salt-tolerant poplar varieties can help expand suitable planting areas and improve the productivity and quality of poplar trees. This, in turn, can contribute to the sustainable development of the forestry industry and related commercial applications, including biofuel production, road greening, and desertification prevention. In conclusion, transgenic poplar overexpressing the *PagSOD2a* gene can significantly mitigate oxidative stress damage induced by salt stress, thereby maintaining better developmental states. These results provide a solid foundation for future efforts in cultivating salt-tolerant tree species and advancing the related commercial and ecological applications.

## Data Availability

The sequencing data are available at https://www.ncbi.nlm.nih.gov/ at NCBI with accession number PRJNA716488.
