# Supplementary material for: PagSOD2a improves poplar salt tolerance by elevating superoxide dismutase activity and decreasing malondialdehyde contents
Source: Front Plant Sci. 2024 Sep 13;15:1456249. doi: 10.3389/fpls.2024.1456249 (PMC11427262; doi:10.3389/fpls.2024.1456249)
Supplement: Supplementary file 2 [file Presentation1.zip › Supplementary_Figures.docx]

Supplementary Material

## Supplementary Figures


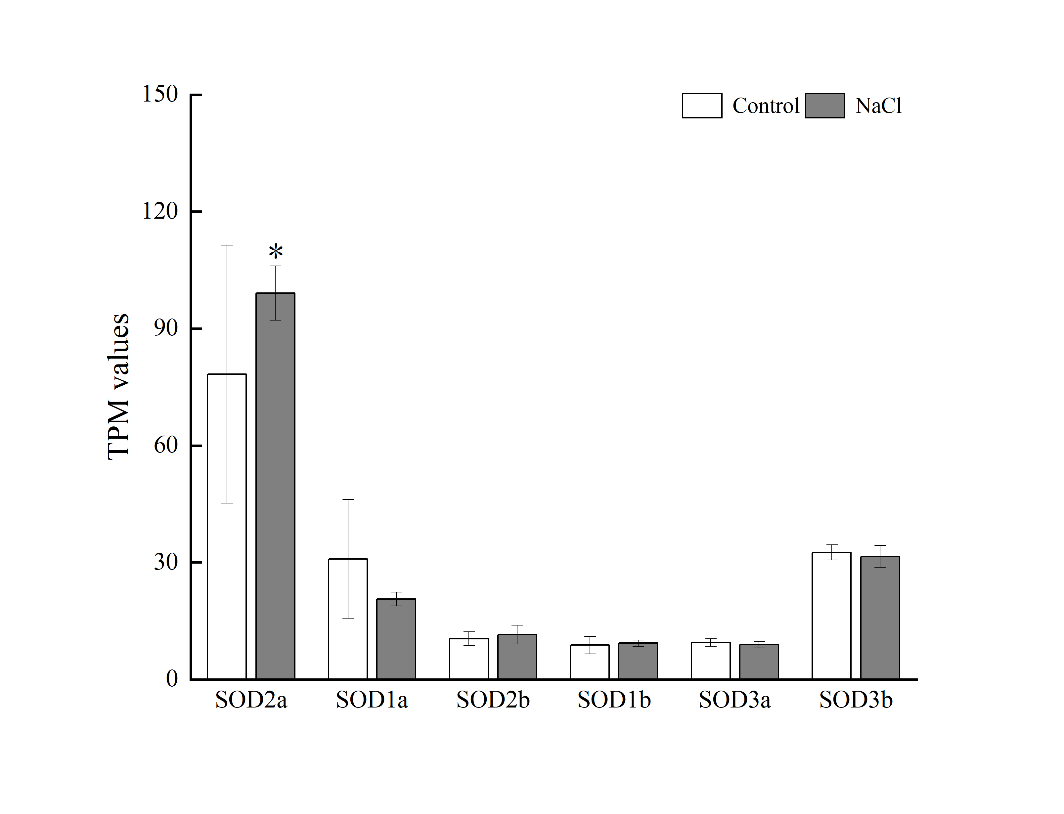


**Supplementary Figure 1** Expression level of six CuZn-SOD genes in poplar leaf under salt stress. * indicate significant differences among conditions (*p*＜0.05). TPM: Transcripts Per Million reads. Control: normal conditions; Salt: Salt treatment conditions.


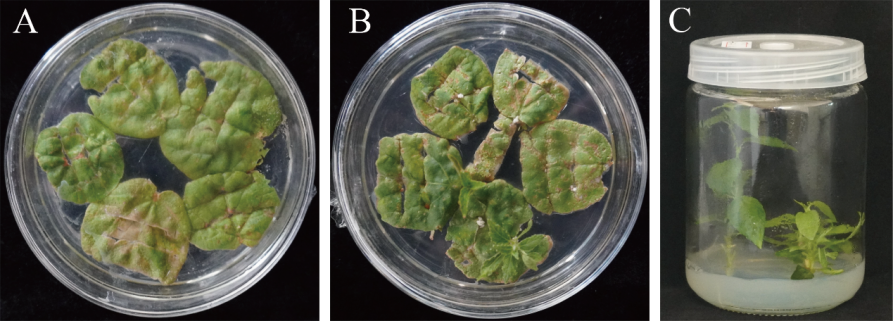


**Supplementary Figure 2** Cultivation of *PagSOD2a* transgenic poplar. (**A**) Infected 84K poplar leaves were cultured on selective medium. (**B**) The leaves with resistant buds grew on the selective medium. (**C**) Resistant buds grew on selective rooting medium.


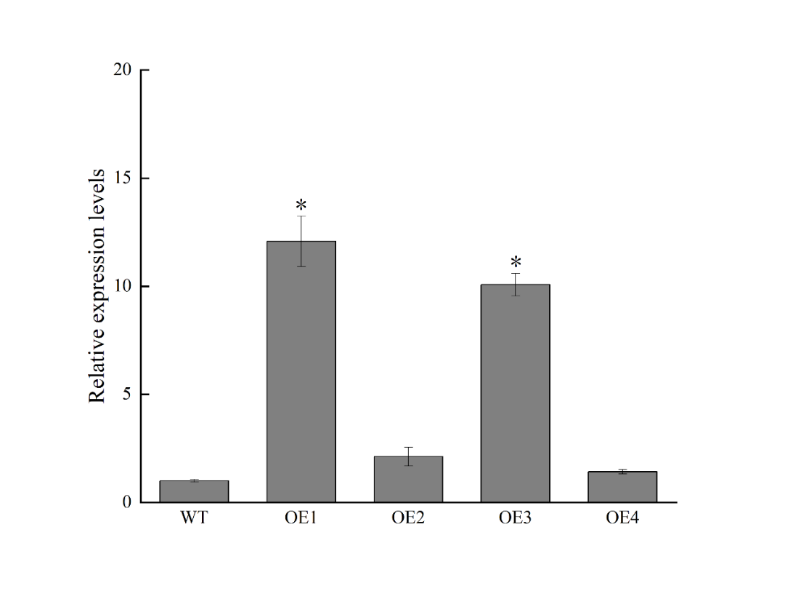


**Supplementary Figure 3** RT-qPCR identification of transgenic poplar. Different lines of pBI121-PagSOD2a transgenic poplar. * indicate significant differences among lines (*p*＜0.05).


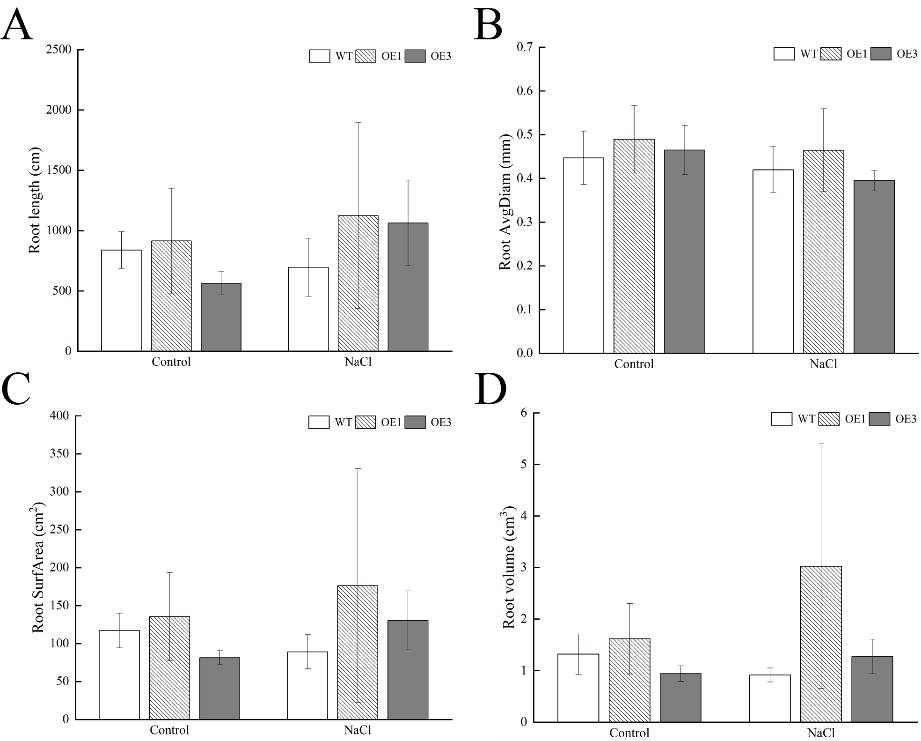


**Supplementary Figure 4** The root morphological indexes of *PagSOD2a* transgenic poplar and non-transgenic poplar under salt treatment and non-salt treatment conditions. (**A**) Root length. (**B**) Root AvgDiam. (**C**) Root SurfArea. (**D**) Root volume. Control: normal conditions; Salt: Salt treatment conditions.
