# Supplementary material for: PagSOD2a improves poplar salt tolerance by elevating superoxide dismutase activity and decreasing malondialdehyde contents
Source: Front Plant Sci. 2024 Sep 13;15:1456249. doi: 10.3389/fpls.2024.1456249 (PMC11427262; doi:10.3389/fpls.2024.1456249)
Supplement: Supplementary file 3 [file Table1.docx]

Supplementary Material

# Supplementary Tables

**Table S1** Primer sequences

| Name | Primers（5′→3′） | Length |
| --- | --- | --- |
| SOD2a-F | GAAGAAGCTCGTCGCACATTATC | 23 bp |
| SOD2a-R | GAACGAGGTGAAAGCAAGCAGC | 23bp |
| SOD2a-121-F | GCGCCCGGGATGCAAGCAGCTGCAATGGCAGC | 32bp |
| SOD2a-121-R | CGCACTAGTTATTGGAGTCAAACCAACAACTC | 32bp |
| SOD2a-q-F | GCACTTCCAGTGTTGAAGGC | 20bp |
| SOD2a-q-R | GCCACTCCATCGGCATTAGC | 20bp |
| pBI121-F | CCATCGTTGAAGATGCCTCTGC | 22bp |
| pBI121-R | CTCTTCGCTATTACGCCAGCTG | 22bp |
| Actin-F | ACCCTCCAATCCAGACACTG | 20 bp |
| Actin-R | TTGCTGACCGTATGAGCAAG | 20 bp |
